# The impact of FAST on the research of fast radio bursts

**DOI:** 10.1093/nsr/nwab204

**Published:** 2021-11-16

**Authors:** Renxin Xu

**Affiliations:** School of Physics and Kavli Institute for Astronomy and Astrophysics, Peking University, China

The Universe has never stopped revealing surprises as science and technology develops. The discovery of fast radio bursts (FRBs) is one such surprise, which are mysterious blips or bursts in the radio band, with a duration of a few milliseconds and large dispersion measures of extragalactic/cosmological origin. Interestingly, a glint was recorded in Australia’s Parkes 64-m telescope in 2001, but was only announced after six years and generally recognized as a new type of cosmic event (rather than being human-made or from aliens) another six years later. The study of FRBs has become one of the focuses of research, especially after the discovery of the first repeating source, FRB 121102, in 2016. Among numerous radio telescopes, it is worth noting that the Canadian Hydrogen Intensity Mapping Experiment (CHIME) is a superb instrument for hunting FRBs, though it was designed to study baryon acoustic oscillation through mapping of redshifted hydrogen.

The CHIME has detected, without doubt, a large number of FRBs due to its unprecedented field of view, but the nature of FRBs is still a matter of debate: are all FRBs repeating? What is the radiative mechanism as well as the physical origin of FRBs? The largest single-dish radio telescope in the world, China’s five-hundred-meter aperture spherical radio telescope (FAST), would be the right tool to solve these problems, taking advantage of its extremely high sensitivity [1]. More and more FRBs, known previously as ‘one-off’ bursts, are identified as repeating ones after comprehensive follow-up surveys with FAST; furthermore, the polarization information tells us more about the radiative mechanism of the extreme events.

FAST, without requiring the complicated data processing necessary for an antenna array, is perfect for pulsar-related sciences, and FRBs, phenomenologically similar to pulsars, are among the targets of top priority [2]. As expected, FAST, in routine operation since 2019, does impact the FRB sciences. FRB 180301 was supposed to be non-repeating, but four bursts from it were detected in July 2019 during work on one of the so-called FAST ‘shared-risk’ projects. Subsequent observations targeted at FRB 180301 discovered more bursts with diversity of the polarization angle features, supporting mechanisms invoking magnetospheres [3]. FAST’s non-detection of radio bursts from galactic magnetar SRG 1935+2154 during its active phase places the most strict upper limit on the fluence, yielding physical conditions that are difficult to satisfy [4]. Recently, FAST scientists have reported a bimodal burst energy distribution of the first discovered repeating FRB, but have showed non-detection of any periodicity or quasi-periodicity for more than one thousand bursts [5]. All these achievements reflect FAST’s great potential for FRB sciences, and one may anticipate more in the future.

It has been about fifteen years since *Science* published Lorimer’s FRB paper, and we understand more now. The underlying physics could essentially be relevant to the inner structure of a neutron star and thus the nature of cold dense matter at supra-nuclear density *if* the pulsar-like magnetosphere is responsible for the extremely coherent emission. In this sense, FRB is the bringer of good news for solving the big question.

***Conflict of interest statement*.** None declared.
